# The effect of combination treatment of CO_2_-laser irradiation and tetracalcium phosphate/dicalcium phosphate anhydrate on dentinal tubules blockage: an in vitro study

**DOI:** 10.1007/s10103-023-03767-5

**Published:** 2023-04-18

**Authors:** Markus Laky, Mane Egelja, Christoph Kurzmann, Brenda Laky, Muazzez Arslan, Hassan Shokoohi-Tabrizi, Xiaohui Rausch-Fan, Andreas Moritz

**Affiliations:** 1 Division of Conservative Dentistry and Periodontology, University Clinic of Dentistry, Medical University of Vienna, Sensengasse 2a, 1090 Vienna, Austria; 2 Core Facility Applied Physics, Laser and CAD/CAM Technology, University Clinic of Dentistry, Medical University of Vienna, Vienna, Austria; 3 Division of Dental Student Training and Patient Care, University Clinic of Dentistry, Medical University of Vienna, Vienna, Austria

**Keywords:** CO_2_-laser, Dentin sensitivity, Dentin desensitizing agents, Tetracalcium phosphate/dicalcium phosphate anhydrate

## Abstract

The aim of this study was the evaluation of the in vitro efficacy of a carbon dioxide (CO_2_) laser, a tetracalcium phosphate/dicalcium phosphate anhydrate (TP/DP) desensitizer and the combination of the desensitizer and additional CO_2_ laser irradiation as a treatment modality for cervical dentin hypersensitivity. A total of 48 dental specimens, prepared from extracted human premolars and molars, were divided into four groups: a control group, a TP/DP desensitizer paste group, a CO_2_ laser (10.600-nm wavelength) group, and a paste and laser group. The specimens were coated with nail varnish except in the marked area and were then immersed in 2% methylene blue dye for 1 h. The specimens were then washed, dried, and cut longitudinally. Thereafter, photos of 40 dentin specimens were taken and evaluated. The area of penetration was assessed and reported as percentage of the dentin surface area. Additionally eight dental specimens were examined with the aid of a scanning electron microscope and evaluated. Significant differences in the penetration depth were found for all experimental groups compared to the control group. The lowest penetration area was detected in the paste-laser group (16.5%), followed by the laser (23.7%), the paste (48.5%), and the control group (86.2%). The combined treatment of the CO_2_ laser and a TP/DP desensitizer was efficient in sealing the dentinal surface and could be a treatment option for cervical dentin hypersensitivity.

## Introduction

Dentin hypersensitivity is a transient oral pain condition on stimulation of exposed dentin. The prevalence of dentin hypersensitivity generally varies depending on the investigational methods. A recently reported prevalence of 25.5% showed that dentin hypersensitivity is a very common disorder [1]. Dentin hypersensitivity is often found in patients with gingival recession, which is a common result of advanced periodontal disease. Dentin hypersensitivity is also frequently associated with toothbrushing [2]. The hydrodynamic theory explains dentin hypersensitivity by an increase in fluid flow and thereby activation of peripheral nerves [3]. Hypersensitive teeth have a wider diameter of exposed dentinal tubules. Thus, treatment modalities often focus on decreasing the diameter of open dentinal tubules [4].

Several desensitizers available on the market to treat dentin hypersensitivity are calcium phosphate containing desensitizers, which have a good biocompatibility and the capability to occlude dentinal tubules [5]. Another, recently introduced desensitizer contains TP/DP, a combination, which can spontaneously convert to hydroxyapatite. Such a chemical reaction happens within seconds after mixing the powder with water by releasing calcium and phosphate ions, which crystallize into layers and, thus, spontaneously build hydroxyapatite [6]. Calcium phosphate containing desensitizers are generally known to reduce dentin permeability and therefore block nerve activity by occluding dental tubules as well as by their good biocompatibility. However, desensitizers as a single clinical treatment option to improve dentin hypersensitivity are impaired by the fact that such agents are removed by daily brushing and pH changes in the oral cavity [7].

The carbon dioxide (CO_2_) laser is highly absorbed by hydroxyapatite [8]. The thermal effects of the CO_2_ laser reduce the water content of the crystallization and may subsequently improve the physical properties [7] and might help in stabilization of the hydroxyapatite on the dentin surface. Indeed, the CO_2_ laser has been shown to have a good sealing effect on dentinal surfaces [9, 10] on one hand, and Moritz et al. [11] showed that the combination of CO_2_ laser with fluoridation treatment resulted in rapid and long-lasting improvements of the clinical situation in dentin hypersensitivity.

Thus, the aim of this investigation was to examine micro-morphological changes and penetration resistance of dentin samples treated with a TP/DP desensitizer paste separate or combined with CO_2_ laser irradiation.

## Materials and methods

### Sample preparation

For this in vitro study, 48 extracted caries-free premolars and molars were used after approval of the Ethics Review Committee of the Medical University of Vienna (1583/2015).

Forty specimens (10 in each group) were used for permeability testing and eight samples (two in each group) for a micromorphologic examination with a scanning electron microscope (S-4500, Hitachi, Tokyo, Japan). Only the cervical third of the roots was used. Two horizontal sections were prepared under water-cooling using a diamond disc (Plexi Flex, 22 mm, Schütz Dental GmbH, Germany), which was mounted on a low speed handpiece. One section was located at the cemento-enamel junction and the second 3 mm apical to the first section. The cementum was removed with a periodontal curette (4L-4R, GC-American, USA). Samples of 3 × 4 mm were obtained from each tooth. The teeth were immersed for 30 s in 1% citric acid solution for smear layer removal, washed with distilled water, and dried with cotton swaps.

The 48 samples were then arbitrary/randomly divided into four groups and the treatment area was marked:

1. Control group (Con)
2. TP/DP paste group (DP)
3. Laser group (L)
4. Desensitizer paste and laser group (DP + L)

The specimens of the control group (Con) received no treatment. For the desensitizer paste-only group (DP), the Teethmate™ Desensitizer (Kuraray Europe GmbH), a paste consisting of tetracalcium-phosphate [(Ca)_4_(PO_4_)_2_O] and dicalcium phosphate [CaHPO_4_], was used and it was prepared according the manufacturer’s instructions. The paste was then applied to the dentin surface with a microbrush for 30 s with hand pressure. Excess paste was removed with an air/water syringe. In the laser only group (L) irradiation was performed using a CO_2_ laser (Opus Duo EC, Lumenis Germany) with 10,600-nm wavelength and 0.65 W in a continuous mode, perpendicular to the dentin surface, with 5-mm defocus distance and a power density of 129.33 W/cm^2^. The laser irradiation was performed according to Moritz et al. [11]. Irradiation was performed six times for 5 s with a 20-s interval for in-between cooling. In the desensitizer paste and laser group (DP + L), desensitizer paste was applied before irradiation. Both treatments were performed the same way as the DP and L groups.

### Dye penetration

All specimens were coated with three layers of nail varnish, while the marked treatment area was left uncoated. Thereafter, they were immersed in an aqueous solution of 2% methylene blue dye for 1 h at room temperature. The samples were then washed, dried with cotton swaps, and cut perpendicular to the root, and each sample was examined.

**Fig. 1:**
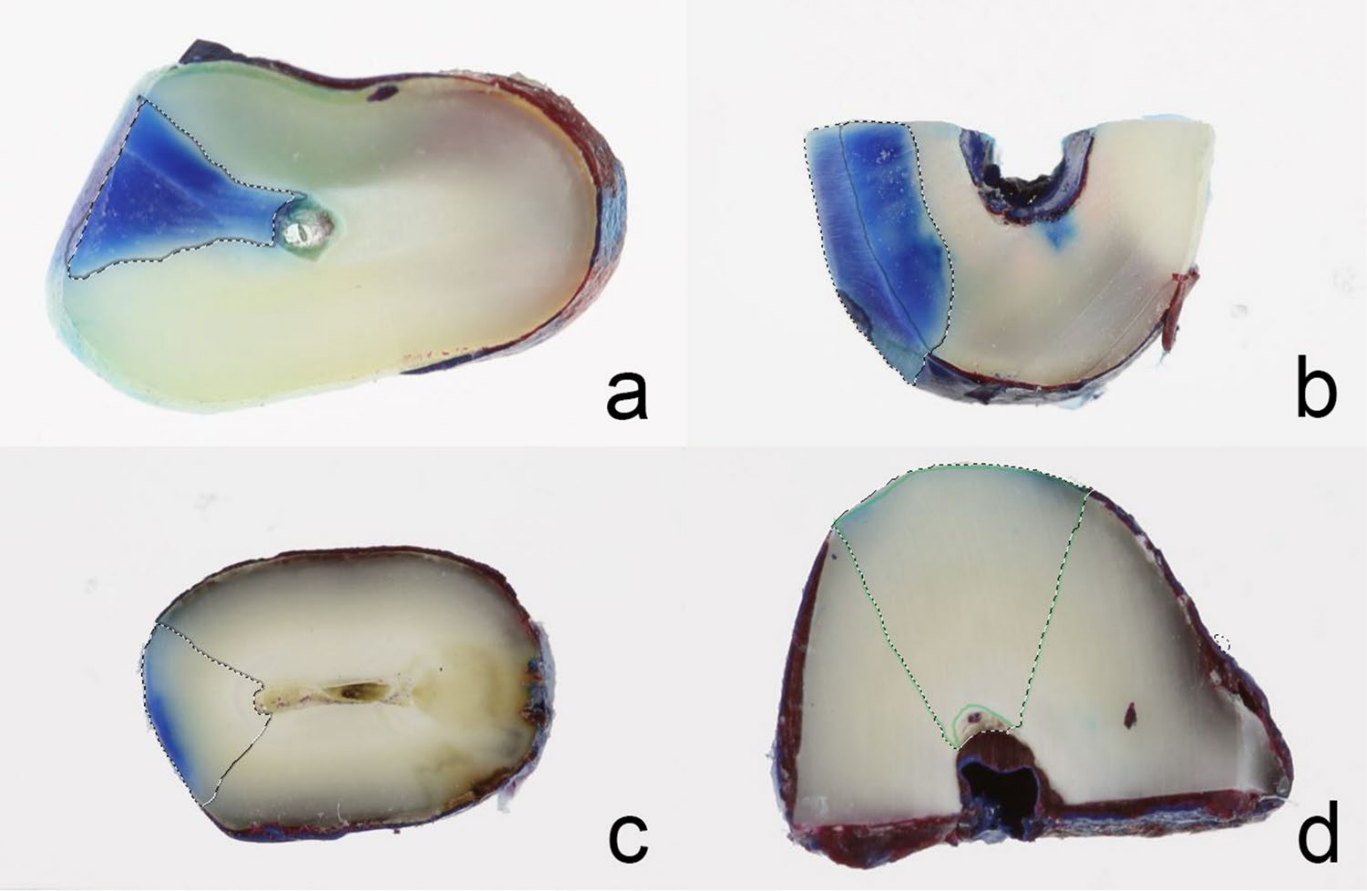
Images showing dye penetration into the dentin. Control (**a**), TP/DP paste (**b**), laser (**c**), laser + TP/DP paste (**d**)

A total of 40 specimens were photographed. The area of dye penetration of the image was evaluated using computer software (Adobe Photoshop CC, version 2015.0.0, Adobe Systems Incorporated). Penetration was measured in pixel from the 2% methylene blue dyed dentin and treatment-marked dentin area (Fig. 1). In two samples of the DL + L group, penetration could not be measured due to insufficient coverage of the sample with the nail polish. The percentage penetration of each sample (*n* = 38) was then calculated by dividing the dyed area by the treatment-marked area multiplied by 100.

### Statistical analysis

Sample size estimation was calculated a priori using G*Power 3.1.9.2 software (Heinrich-Heine-University of Düsseldorf, Düsseldorf, Germany). Using an effect size from our previous investigations (partial eta squared = 0.8) with α = 0.05 and a power of 80%, a sample size of 8 specimens in each group was calculated. For possible dropouts, the number per group was increased to 10. Assumptions of normality (Kolmogorov–Smirnov test, *P* > 0.05) were met across the data set. The Levene test yielded no statistical significance (*P* < 0.05) indicating that assumptions of homogeneity in the data set were met. Data were statistically analyzed using one-way analysis of variance (ANOVA) with Tukey post hoc tests. All data were analyzed using SPSS version 23 (IBM, Chicago, IL, USA). Statistical significance was set at *P* < 0.05.

## Results

**Fig. 2:**
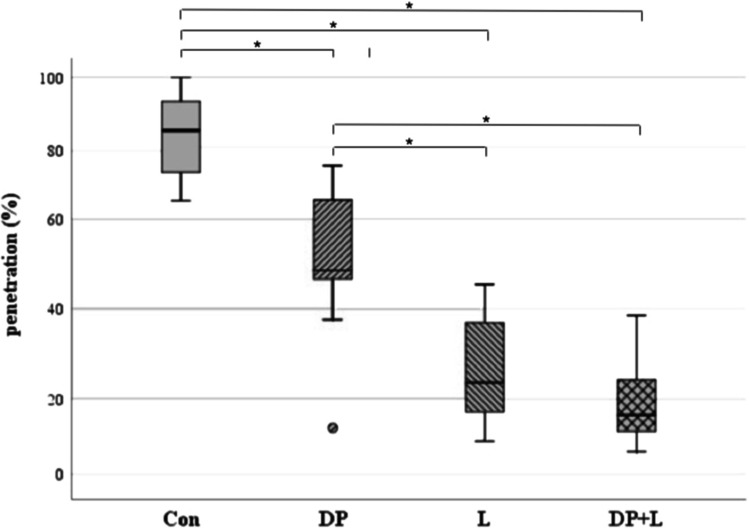
Boxplot showing penetrations of the control (Con), desensitizer paste (DP), laser (L), and desensitizer paste plus laser group (DP + L) group. * < 0.001

A comparison of the penetration between the four groups was significant (*P* < 0.001). Furthermore, the control compared to the treatment groups (DP, L, and DP + L) showed significant differences. The highest penetrations were detected within the control group, followed by the DP and the L group, while the lowest penetration was found in the DP + L group. Penetration was also significantly different between DP and L as well as with DP + L, but not between L and DP + L. All penetration data are presented in Table 1 and Fig. 2.

**Table 1:** Comparisons of penetration (%) between the groups

Penetration (%)	Control (Con)	Desensitizer paste (DP)	Laser (L)	DP + L
N	10	10	10	8
Mean ± SD	84.3 ± 11.9	50.3 ± 18.0	25.4 ± 12.6	19.2 ± 10.4
Median (min–max)	86.2 (65.7–100.0)	48.5 (10.3–74.3)	23.7 (7.4–45.4)	16.4 (7.9–38.5)
*P-value* (vs. Con)	–	< 0.001	< 0.001	< 0.001
*P-value* (vs. DP)	–	–	0.001	< 0.001
*P-value* (vs. L)	–	–	–	0.774

*Max* maximum, *Min* minimum, *SD* standard deviation

**Fig. 3:**
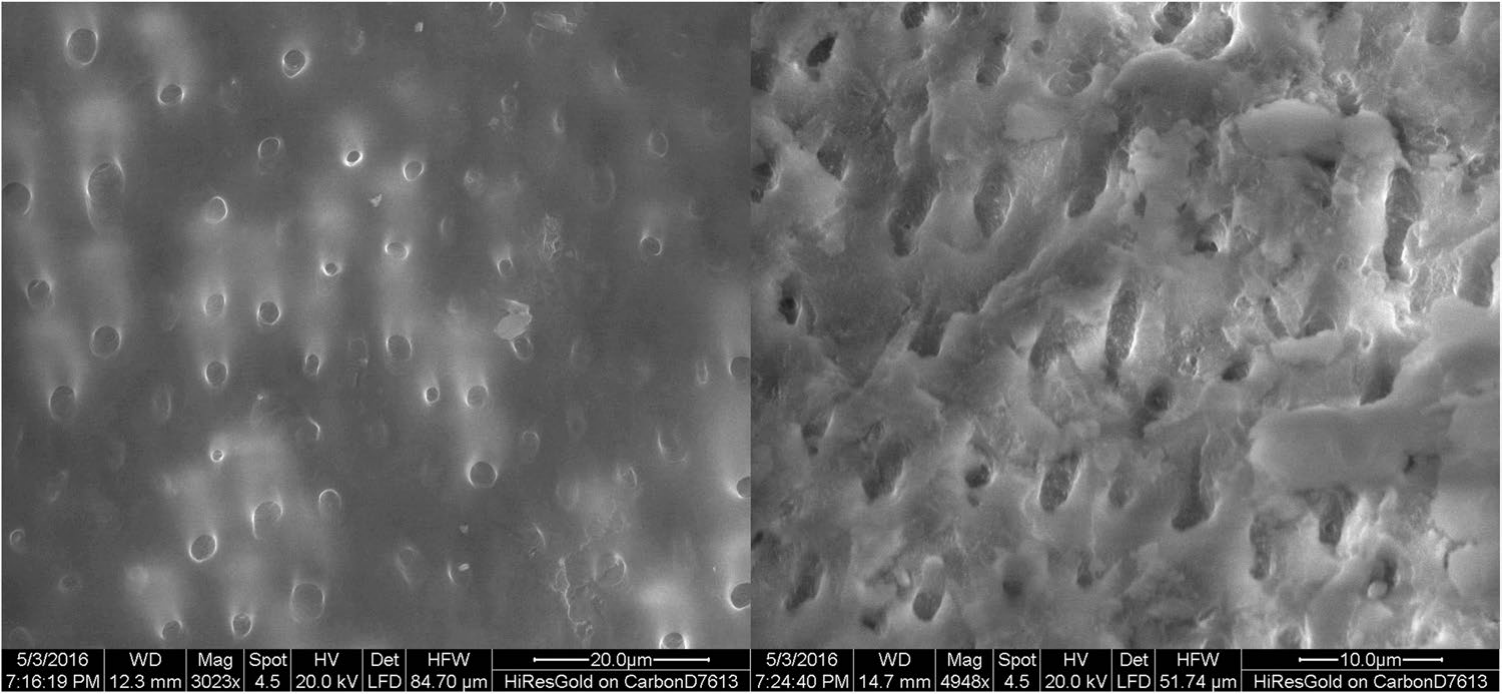
SEM images of the control group showing the typical morphology of the dentin surface after removal of the smear layer. The dentin tubules are opened

**Fig. 4:**
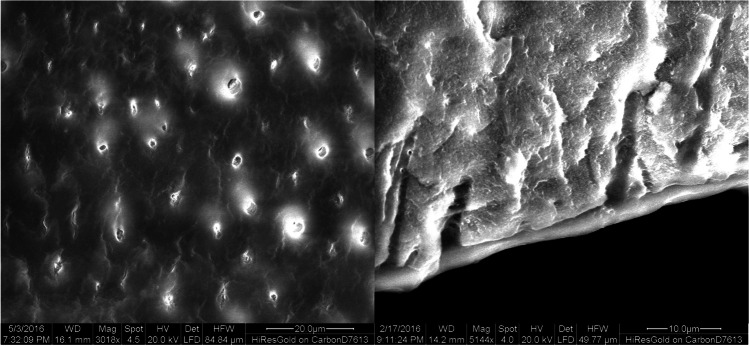
SEM images of the desensitizer paste (DP) group showing partially and completely closed dentin tubules. The TP/DP desensitizer layer has formed on the surface

**Fig. 5:**
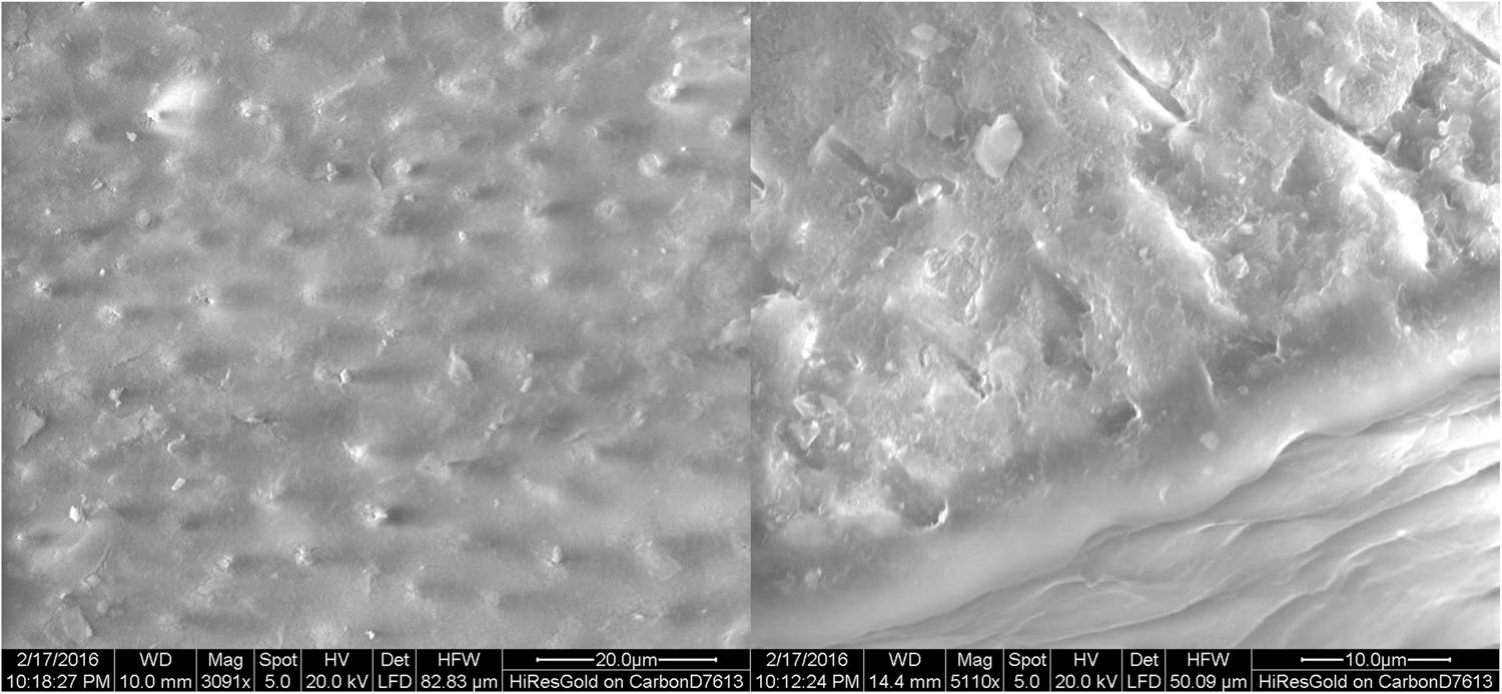
SEM images of the laser group showing partially and completely closed dentin tubules. In contrast to the desensitizer paste-only group, the dentinal tubules orifices are narrowed

**Fig. 6:**
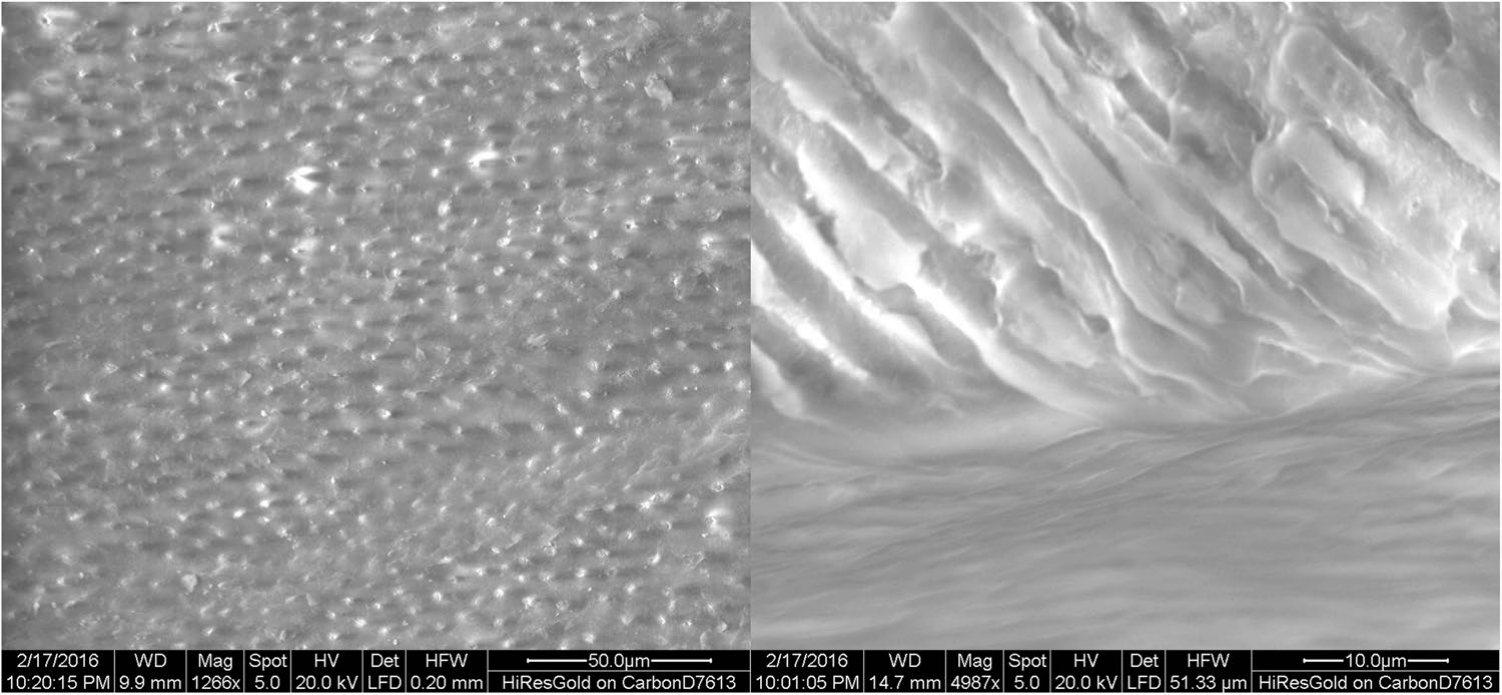
SEM images of the desensitizer paste plus laser group (DP + L) showing predominantly completely closed dentin. The combined application of DP and L showed that DP clogged the dentinal tubules orifices and the subsequent CO_2_ laser treatment fused both the dentin and the DP

Scanning electron microscopy (SEM) observations showed open dentinal tubules with no smear layer in the control group (Fig. 3). Partially and completely closed dentin tubules were detected in the DP (Fig. 4) and L (Fig. 5) group, while closed dentin tubules dominated in the DP + L group (Fig. 6). In contrast to the DP-only group, the dentinal tubule orifices were narrowed due to the peritubular dentin fused by CO_2_ laser treatment in the L group on one hand; while in contrast to groups DP and L, in the combined group (DP + L), the application of TP/DP desensitizer clogged the dentinal tubules orifices first and subsequently the CO_2_ laser treatment fused both the dentin and the desensitizer.

## Discussion

The results showed that the application of a TP/DP desensitizer paste occluded dentinal tubules and decreased dye penetration into the dentin. However, sealing of the dentinal tubules was significantly increased by CO_2_ laser irradiation with 0.65 W, 6 times for 5 s in each cycle with a 20-s break between the cycles. The best results regarding penetration testing yielded the combination of the TP/DP paste and CO_2_ laser irradiation.

TP/DP is transformed in an aqueous environment to hydroxyapatite, the main mineral in enamel and dentin [12]. The mechanism of transformation involves dissolution of calcium and phosphate ions from TP/DP powder, which then precipitate as hydroxyapatite on the surface of the particles in the mixture. The mixing of the two components provided a paste, which could penetrate into the dentinal tubules [13]. The occluding effect on dentinal tubules could be seen by SEM with a magnification of × 3000 and additionally resulted in a significant, immediate reduction of dye penetration compared to control specimens. Ishihata et al. [12] also reported an immediate and lasting reduction in dentin disc permeability. Hydraulic conductance after the application of TP/DP was significantly reduced up to 1 month.

Unlike the radiation of the Nd:YAG laser, which is absorbed especially by pigmented tissues, the infrared radiation of the CO_2_ laser is absorbed near the surface particularly by tissues containing calcium and phosphate [10]. The thermal effect of the laser removes the water of the crystallization and subsequently is able to improve physical properties of the calcium phosphate crystals [7]. Surface changes by laser irradiation on the newly formed hydroxyapatite layer resulted in a tendency for an additional decrease of dye penetration. Moritz et al. showed that CO_2_ laser irradiation resulted in nearly complete closure of the dentinal tubules in the hypersensitive dental neck region [11]. In this study, this could also be seen with SEM imaging and resulted in a considerably reduced dye penetration.

The CO_2_-laser irradiation is characterized by a low tissue penetration depth, a high absorption in water, and in hydroxyapatite. Gholami et al. [14] reported a 42.3% decrease of the mean tubular diameter via changes of the peritubular dentin and, thus, concluded that CO_2_ laser energy, because of the high absorption in hydroxyapatite, could effectively lead to dentin recrystallization [14]. Hypersensitive teeth showed an eight times increased number of open tubules per area than non-sensitive teeth, and tubules’ diameter were about two times larger than in non-sensitive teeth [15, 16]. It was concluded that in non-sensitive dentin, almost all dentinal tubules were closed. Sensitive radicular dentin has a substantial number of exposed open dentinal tubules on the surface. Hydraulic movement of the tubular fluid in exposed dentin is a possible main trigger of direct stimulation of pulpal mechanoreceptors [17].

In the treatment of dentin hypersensitivity, lasers could on one hand result in an occluding effect of dentinal tubules or on the other hand lead to desensitization by changing or reducing the pulpal nerve’s pain threshold [18]. A reduction of the pain threshold is to be expected especially for laser wavelengths with a low absorption in dentinal tissue like diode lasers [19]. Several lasers have been reported in the literature as effective devices to reduce tooth sensitivity [14].

Several dentinal tubule sealants such as arginine, resin bonding agents, and cavity varnishes and also potassium nitrate and fluoride varnishes, which are interacting with the electrical activity of nerves, are successful in reducing hypersensitivity. However, most of the methods offer only temporary and unpredictable desensitization [14]. Therefore, new and innovate treatment options are still needed, especially to enhance long-term clinical results.

Despite the fact that this in vitro study on dentinal hypersensitivity showed that surface changes are possible and that desensitizer paste and CO_2_ laser irradiation are able to occlude dentinal tubules, results have to be interpreted with caution. Further studies have to determine the clinical effectiveness of the different surface treatments in dentin hypersensitivity. The effect of both methods, desensitizer paste and laser irradiation, are believed to last longer than other dentinal tubule sealants (such as arginine or fluoride varnishes) [20]; however, long-term in vivo effects of the investigated procedures are still unknown. Negative effects like a charred dentinal surface with craters and cracks were reported for the CO_2_ laser [17]. These laser adverse effects are dependent on the energy level and may be caused by irregularities in the dentin structure resulting in areas of high absorption for the laser irradiation and subsequent deleterious outcomes.

The application of TP/DA resulted in less dye penetration into the dentin and a partial occlusion of dentinal tubules. However, the additional use of a CO_2_ laser was more efficient in sealing the dentinal surface. The application of TP/DA and laser irradiation showed a further trend in reducing dye penetration and an additional effect in the sealing capability could be seen.

## Conclusion

The combined treatment of dentin with a TP/DP desensitizer paste and a CO_2_ laser was efficient in sealing the dentinal surface and, thus, could be a treatment option for cervical dentin hypersensitivity.
